# Preparation and Utilization of a Highly Discriminative Absorbent Imprinted with Fetal Hemoglobin

**DOI:** 10.3390/polym16192734

**Published:** 2024-09-27

**Authors:** Ka Zhang, Tongchang Zhou, Cedric Dicko, Lei Ye, Leif Bülow

**Affiliations:** Division of Pure and Applied Biochemistry, Department of Chemistry, Lund University, Box 124, 22100 Lund, Sweden; ka.zhang@tbiokem.lth.se (K.Z.); tongchang.zhou@tbiokem.lth.se (T.Z.); cedric.dicko@tbiokem.lth.se (C.D.); lei.ye@tbiokem.lth.se (L.Y.)

**Keywords:** fetal hemoglobin, adult hemoglobin, molecular imprinting, purification, polymer

## Abstract

Development in hemoglobin-based oxygen carriers (HBOCs) that may be used as alternatives to donated blood requires an extensive supply of highly pure hemoglobin (Hb) preparations. Therefore, it is essential to fabricate inexpensive, stable and highly selective absorbents for Hb purification. Molecular imprinting is an attractive technology for preparing such materials for targeted molecular recognition and rapid separations. In this case study, we developed human fetal hemoglobin (HbF)-imprinted polymer beads through the fusion of surface imprinting and Pickering emulsion polymerization. HbF was firstly covalently coupled to silica nanoparticles through its surface-exposed amino groups. The particle-supported HbF molecules were subsequently employed as templates for the synthesis of molecularly imprinted polymers (MIPs) with high selectivity for Hb. After removing the silica support and HbF, the resulting MIPs underwent equilibrium and kinetic binding experiments with both adult Hb (HbA) and HbF. These surface-imprinted MIPs exhibited excellent selectivity for both HbA and HbF, facilitating the one-step isolation of recombinant Hb from crude biological samples. The saturation capacities of HbA and HbF were found to be 15.4 and 17.1 mg/g polymer, respectively. The present study opens new possibilities for designed resins for tailored protein purification, separation and analysis.

## 1. Introduction

Molecular imprinting, i.e., the preparation of artificial recognition sites in synthetic polymers, is rapidly gaining popularity in a wide field of applications in biotechnology, biochemistry and pharmaceutical research [1,2,3,4,5]. Due to their chemical and physical stability, facile synthesis and low fabrication cost, molecularly imprinted polymers (MIPs) have been widely evaluated for the separation and detection of particularly low molecular weight compounds [6,7,8,9,10]. Since the preparation of MIPs against high molecular weight biologics still largely remains a challenge, large biological entities like proteins, DNAs, cells and viruses have often been considered to be upcoming areas for molecular imprinting. The inherent properties of these targets in terms of size, complexity and conformational instability have been the main hindrances for developing useful MIPs in these cases [11,12]. In spite of the many challenges, numerous techniques have been devised to create protein-imprinted polymers. The recent advancements in this domain have been significant and critically evaluated in several excellent review articles [2,3,13,14,15].

Among some alternative options, surface imprinting techniques have most often been used for protein imprinting, which is achieved by either synthesizing a thin polymer film or by affixing the protein template onto the substrate surface and then following up with polymerization around it [16,17,18]. Surface imprinting solves the issues of restricted mass transfer in the obtained material and facilitates removal of the template. In our recent studies, we have developed a new procedure for preparing protein imprinted polymers with binding sites on the surface of densely cross-linked polymer beads through Pickering emulsion polymerization [19]. In a Pickering emulsion, solid particles are responsible for stabilizing dispersed liquid droplets, unlike traditional surfactants. The fundamental concept behind this droplet stabilization is the distribution of solid particles between the two immiscible liquids [20]. Due to its exceptional stability and the ability to be formed with cost-effective and non-toxic particles, Pickering emulsion has found applications in various fields [21,22,23]. Although the utilization of Pickering emulsion in molecular imprinting is a relatively recent advancement, it holds significant potential for the creation of imprinted polymers. directed against predefined molecular targets [24,25,26,27,28].

Herein, we demonstrate the use of Pickering emulsions to fabricate fetal hemoglobin (HbF)-imprinted polymer beads. Hb is a protein present in red blood cells (RBCs) primarily responsible for transporting oxygen from the lungs to the body’s tissues [29]. In humans, Hb exhibits heterogeneity, with various variants and derivatives. The most prevalent human Hb, known as human adult Hb (HbA), is a tetrameric protein comprising an (αβ)_2_ homodimer. Human fetal hemoglobin (HbF) is the primary Hb in fetal erythrocytes, appearing during the last seven months of development in the uterus and persisting for up to six months after birth [30]. HbF shares α subunits with HbA but substitutes β subunits with γ subunits. Notable Hb variants of significance include HbC, HbE and HbS, and their separation in clinical settings is achieved by electrophoretic techniques [31,32]. In recent years, challenges such as a shortage of blood donors, the limited shelf life of blood products, a growing elderly population and concerns related to the transmission of pathogens like HIV have gained increasing attention and have triggered the development of blood substitutes [33,34,35,36]. Among them, Hb-based oxygen carriers (HBOCs) have attracted much attention and been studied extensively over the years [37,38,39,40,41]. However, HBOCs are still at an early stage of clinical development, and we still need to develop new technologies to characterize and purify these biological agents.

**Scheme 1:**
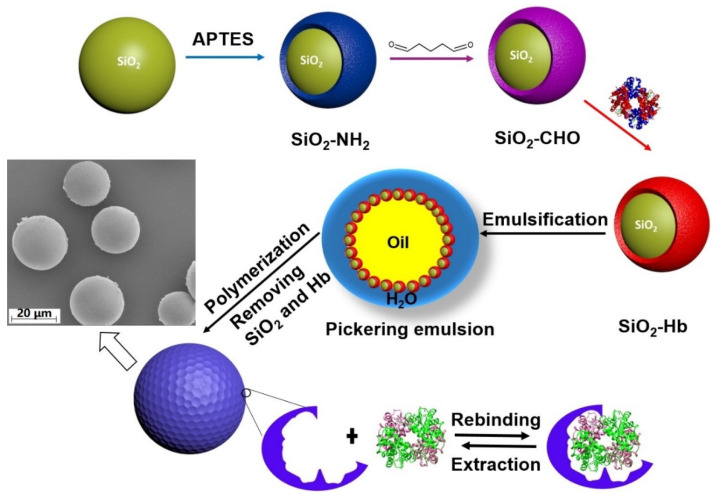
Schematic illustration of the MIP particle preparation using the Pickering emulsion technique. HbF-immobilized silica particles were used to stabilize the Pickering emulsion. Following the polymerization, the solid beads were collected and subjected to washing. The morphology of the obtained MIP was also checked by SEM.

In this study, we have focused on designing MIPs against HbF, since this is an attractive source of Hb for HBOC production [39]. The preparation of the HbF-imprinted polymers is achieved through a multistep procedure (Scheme 1). In this approach, HbF was firstly linked onto the surface of silica nanoparticles. During the Pickering emulsion polymerization process, the protein served as a template and facilitated the formation of accessible binding sites on the resulting surface of the polymer beads. The supporting silica particles and proteins were subsequently removed by an acid treatment followed by extensive washings. The molecular separation performance of the HbF-imprinted polymer beads was characterized, and the prospects of employing these robust molecularly imprinted polymers (MIPs) as a chromatographic resin for the direct and specific isolation of Hb from crude cellular samples were also examined.

## 2. Materials and Methods

### 2.1. Materials

Silica nanoparticles (diameter 10 nm), 3-aminopropyltriethoxysilane (APTES), glutaraldehyde (25% in water), acrylamide (Am, ≥98%) and ethylene glycol dimethacrylate (EGDMA, 98%) were purchased from Sigma-Aldrich (St. Louis, MO, USA). Merck (Darmstadt, Germany) supplied methacrylic acid (MAA, 98.5%), 2,2′-azobis(2-methylpropionitrile) (AIBN, 98%) and sodium dodecyl sulphate (SDS, 98%), while other solvents and inorganic salts, of analytical reagent grade standards, were utilized without additional purification. The polyacrylamide gels (4–20%, Mini-Protean 3) for SDS-PAGE analysis were procured from Bio-Rad (Hercules, CA, USA).

### 2.2. Protein Samples

In order to produce recombinant human HbA and HbF, the optimized genes were inserted into the pET-Duet-1 expression plasmid (Novagen, Madison, WI, USA) and expressed in *E. coli* BL21 (DE3) as described earlier [42]. Bacterial lysis was achieved through sonication, and the soluble protein fraction underwent initial purification via cation exchange chromatography (CM-Sepharose, Cytiva, Uppsala, Sweden), followed by subsequent purification using a strong anion exchanger (Q Sepharose HP, Cytiva, Uppsala, Sweden) [43,44]. A green fluorescent protein-HbF (GFP-HbF) fusion protein was produced by genetically linking GFP to the N-terminus of the α chain HbF. To further simplify the production of this protein, the α and γ chains of HbF were also linked by twelve amino acid residues (GGS)_4_ to generate a single polypeptide chain. This reporter fusion protein was used for characterizing the binding properties of the MIP-HbF particles. GFP-HbF was expressed in *E. coli* BL21 (DE3) cells and was purified using two chromatographic steps. The crude bacterial extracts were firstly loaded to a Ni^2+^-based Immobilized Metal ion Affinity Chromatography (IMAC) FF column equilibrated with a loading buffer (20 mM sodium phosphate and 10 mM imidazole, pH 8.0). For the elimination of host cell proteins, the column was rinsed with a buffer identical to the one used but supplemented with 20 mM imidazole and 0.2 M NaCl. Elution was achieved using 20 mM sodium phosphate, pH 8.0, supplemented with 350 mM imidazole and 0.2 M NaCl. HbF containing fractions were subsequently dialyzed and changed to 20 mM Tris-HCl buffer, pH 8.3. It was then applied to a strong anion exchanger (Q Sepharose HP, GE Healthcare) that had been equilibrated with the same buffer. The bound protein was subsequently eluted using a linear gradient, extending to 50 mM sodium phosphate buffer at pH 7.2, with the addition of 100 mM NaCl. The final Hb sample was collected and concentrated using a Viva-Spin column with a 30,000 MWCO (Sartorius, Gottingen, Germany). The concentrated protein solution was rapidly frozen in liquid nitrogen and stored at −80 °C until further characterization. Throughout the handling process, the Hb protein was consistently maintained in its CO form to avoid intrinsic oxidative side-reactions associated with hemoglobin. The Hb concentration was determined by spectroscopy (Agilent Cary 60 UV–Vis Spectrophotometer, Santa Clara, CA, USA) at 419 nm (ε_419_ = 191 mM^−1^ cm^−1^).

### 2.3. Preparation of HbF-Imprinted Polymers Using Immobilized Templates

To firstly prepare the aldehyde-modified silica nanoparticles, 100 mg silica nanoparticles were suspended in 10 mL toluene by sonication. Then, 100 μL APTES was added slowly and then was left to incubate at ambient temperature for 4 h under stirring. The obtained particles (SiO_2_-NH_2_) were collected by centrifugation and rinsed repeatedly by pure water before dispersed in 20 mM phosphate buffer, pH 6.8. To graft aldehyde groups, 50 μL glutaraldehyde was added to the suspended particles, and the reaction mixture was agitated at room temperature for a duration of 12 h. Subsequently, the aldehyde-modified silica nanoparticles (SiO_2_-CHO) were collected, rinsed with water and, in a final step, dried in a depressurized desiccator.

In a round-bottom flask, 10 mg SiO_2_-CHO nanoparticles were added into 3 mL HbF solution (3 mg/mL, 20 mM phosphate buffer, pH 6.8). The mixture was stirred at room temperature for 2 h. The supernatant was then removed by centrifugation, and the HbF-immobilized silica particles were obtained. It was estimated that approximately 2.5 mg HbF was immobilized on the silica (0.25 mg HbF/mg silica, estimated from the HbF in the supernatant).

The HbF-immobilized silica particles were then dispersed in a test tube with 3 mL 20 mM phosphate buffer, pH 6.8, phosphate, followed by adding a mixture of 100 mg of Am, 136 μL of MAA and 250 μL of 3 M NaOH. For the oil phase, a combination of 100 μL of toluene, 400 μL of EGDMA (2.1 mmol) and 10 mg of AIBN was mixed. Subsequently, the two phases were vigorously combined to create a stable Pickering emulsion. The test tube was sealed, and polymerization was initiated at 70 °C, allowing it to proceed for 16 h. After polymerization, the solid beads were collected and subjected to two rounds of methanol washing. The removal of silica particles was achieved using a solution consisting of 30 mL of methanol and 1 mL of 30% HF. The remaining polymer beads were then thoroughly rinsed with 10% acetic acid and 5% SDS solutions. Finally, the polymer beads were washed with water and dried under reduced pressure in a desiccator.

### 2.4. Surface Characterization of the MIP Beads

To explore the surface characteristics of the silica nanoparticles, the dried particles were transferred onto the sample plate of an FTIR instrument (Nicolet iS5, Thermo Scientific, Madison, WI, USA) and analyzed directly. All spectra were collected at ambient temperature in the range 4000–500 cm^−1^ with 16 scans. In addition, a scanning electron microscope (Thermal Field Emission SEM LEO 1560, Zeiss, Oberkochen, Germany) was employed for the examination of the surface morphology of the MIP beads in a protein-binding analysis.

To investigate the adsorption equilibrium of the MIP particles, 5 mg of imprinted polymer was added into 1 mL 20 mM sodium phosphate buffer, pH 6.0, containing different amounts of HbA/HbF. The mixture was gently agitated at 4 °C for a duration of 10 min, followed by centrifugation at 13,000 rpm for 2 min. The unbound concentration of Hb in the supernatant was quantified spectrophotometrically at 419 nm using a Cary 60 spectrophotometer (Agilent technologies, Santa Clara, CA, USA). The Hb adsorption capacities (Q) of the particles were calculated by the following equation:

(1) $$Q_{eq}=\frac{\left(C_{0}-C_{t}\right)\times V}{m}$$

In the above equation, *C*_0_ (μM) is the initial concentration of the protein solution, *C_t_* (μM) is the final protein concentration of the supernatant solution, *V* (mL) is the volume of the initial solution and *m* (mg) is the mass of the particles. The adsorption equilibrium was then fitted to the Langmuir equation:

(2) $$\frac{C_{eq}}{Q_{eq}}=\frac{C_{eq}}{Q_{m}}+\frac{1}{k_{a}Q_{m}}$$

where *Q_eq_* is the equilibrium adsorbed amount of Hb, *Q_m_* is the saturation capacity, *C_eq_* is the equilibrium concentration and *k_a_* is the adsorption equilibrium constant.

Similarly, the adsorption kinetics experiments were performed with an initial Hb concentration of 6 μM, and other factors were the same as in the equilibrium binding test. Samples were taken at regular time intervals for spectroscopic analysis to determine the Hb concentration. The absorption data were then fitted to a pseudo-second-order kinetic model:

(3) $$\begin{array}{l} \frac{t}{Q_{t}}=\frac{1}{v}+\frac{1}{Q_{eq}}t \end{array}$$

in which *ν* is the initial sorption rate, and *Q_t_* and *Q_eq_* are the amounts of Hb adsorbed at time *t* and at equilibrium, respectively. By plotting *t/Q_t_* against *t*, the values of *v* and *Q_e_* can easily be obtained.

### 2.5. Competitive Protein-Binding Assay

To investigate the disparity in binding between HbA and HbF to the MIP particles, 5 mg of imprinted polymers were combined with 1 mL of 20 mM sodium phosphate buffer at pH 6.0 containing 4 μM GFP-HbF and various concentrations of HbA/HbF (ranging from 0 to 5 μM). The mixture was gently stirred at 4 °C for 10 min, followed by centrifugation at 13,000 rpm for 2 min. The concentration of free GFP-HbF in the supernatant was determined using a PTI Quanta master spectrofluorometer (Photon Technology International, Lawrenceville, NJ, USA), with excitation and emission wavelengths set at 390 nm and 508 nm, respectively. Additionally, UV–Visible spectra were recorded to correct for the inner filter effect in the fluorescence emission spectra using an appropriate equation:

$$F_{corr}=F_{obs}\times 10^{\frac{A_{exc}+A_{em}}{2}}$$

where *F_corr_* and *F_obs_* are the corrected and uncorrected fluorescence intensities, and *A_exc_* and *A_em_* are the absorbance values at the current excitation and emission wavelengths. The amount of GFP-HbF bound to the polymer particles was calculated by subtracting the free GFP-HbF from the total GFP-HbF added.

### 2.6. Selective Capture of Hb from Recombinant HbA and HbF Complex Samples

Following purification, the purified recombinant HbA/HbF is routinely flash-frozen in liquid nitrogen and stored at −80 °C. However, various biophysical changes can occur in Hb samples during freezing, storage and subsequent thawing. To assess the selectivity of the MIP particles, 5 mg of imprinted polymer beads were added to 1 mL of 20 mM sodium phosphate buffer at pH 6.0 containing different amounts of thawed recombinant HbA/HbF samples. The mixture was gently stirred at 4 °C for 10 min, then centrifuged at 13,000 rpm for 2 min. The concentration of free Hb in the supernatant was measured spectroscopically, and the different protein solutions were visualized by SDS-PAGE on precast 4–20% Mini-Protean TGX gels (Bio-Rad, Richmond, CA, USA) stained with Coomassie brilliant blue.

### 2.7. Chromatographic Analysis

The Hb-imprinted particles, having been prepared beforehand, were carefully loaded into a HR 5/5 column from GE Healthcare in Uppsala, Sweden, for chromatographic analysis. All chromatographic procedures were conducted utilizing an ÄKTA Avant system also from GE Healthcare, controlled by Unicorn software version 7.0. Initially, the MIP column was equilibrated by rinsing the particles with a 20 mM sodium phosphate buffer at pH 6.0. Subsequently, protein samples were injected into the column under the same buffer conditions. Elution was accomplished through a linear gradient, transitioning to a final buffer of pH 8.0 at a flow rate of 1 mL/min. The fractions containing Hb were then gathered and concentrated using a Vivaspin column with a 30,000 MWCO (manufactured by Sartorius). The efficacy of Hb purification was assessed by visualizing the samples through SDS-PAGE, employing precast 4–20% Mini-Protean TGX gels from Bio-Rad in Richmond, CA, USA, and staining them with Coomassie brilliant blue.

## 3. Results

### 3.1. Preparation and Characterization of HbF-MIPs

The process for synthesizing HbF-MIPs is outlined in Scheme 1. In prior research, we devised a method for creating MIPs targeting HbA [19]. HbA was initially physically adsorbed onto the silica surface, but this interaction may be disrupted during MIP synthesis, for instance, through changes in pH or the introduction of chemicals. Thus, precise control over the conditions during polymerization is essential to prevent the potential leakage of Hb. Additionally, it is crucial to ensure the stability of the oil-in-water emulsion. In this study, we opted to covalently attach HbF to the silica surface via surface-exposed amino groups. Initially, the silica surface was modified with a monolayer of the silanizing agent APTES to introduce amino groups onto the surface. Subsequently, the amino-functionalized silica (SiO_2_-NH_2_) was treated with glutaraldehyde to activate the aldehyde-modified silica nanoparticles (SiO_2_-CHO). Finally, 2.5 mg of HbF was covalently bonded to the surface of 10 mg of SiO_2_-CHO through the formation of an imine bond between the aldehyde groups on the silica particles and the amino groups on the protein.

**Figure 1:**
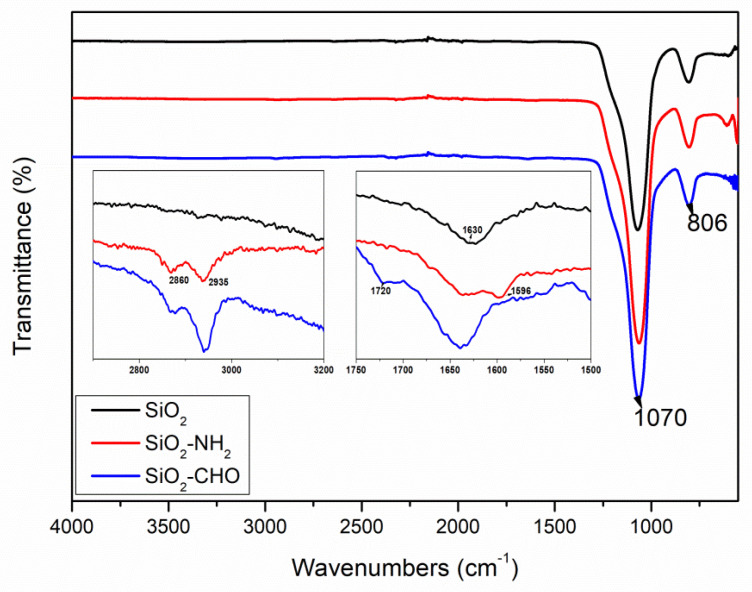
FTIR spectra of silica nanoparticles with different functional groups.

Figure 1 displays the FTIR spectra of the silica nanoparticles before and after modification. All samples exhibited strong stretching vibrations of the Si-O-Si bond at around 1070 cm^−1^ and Si-O bending vibrations at 806 cm^−1^ [45,46]. Peaks near 1630 cm^−1^ were assigned to stretching vibrations of the surface silanol groups in the silica matrix. Following the introduction of amino functionality, SiO_2_-NH_2_ displayed characteristic peaks of the N-H bending vibration at 1596 cm^−1^. Compared to SiO_2_-NH_2_, the spectrum of SiO_2_-CHO showed the complete disappearance of the N-H band at 1596 cm^−1^ since we used an excess of glutaraldehyde. A new band also appeared at 1720 cm^−1^ due to the C=O bond from the aldehyde group. In the spectra of SiO_2_-NH_2_ and SiO_2_-CHO, new peaks emerged near 2860 and 2935 cm^−1^, attributed to C-H absorption. These peaks indicate the successful completion of each grafting step and the successful grafting of aldehydes onto the surface of the silica. Additionally, it was observed that the SiO_2_-CHO nanoparticles exhibited a pink coloration.

Subsequently, the HbF-immobilized silica nanoparticles served as stabilizing agents to form an oil-in-water Pickering emulsion. The oil phase was then polymerized through free radical initiation. Following the removal of the silica nanoparticles and the template protein, HbF-imprinted MIP beads with high rigidity and specific binding sites on the surface were obtained. Characterization of the MIPs was conducted via SEM (refer to the image in Scheme 1), revealing that the average size of the Hb-MIP beads was approximately estimated to be 20 ± 5 μm.

### 3.2. Molecular Recognition Property of HbF-MIPs

**Figure 2:**
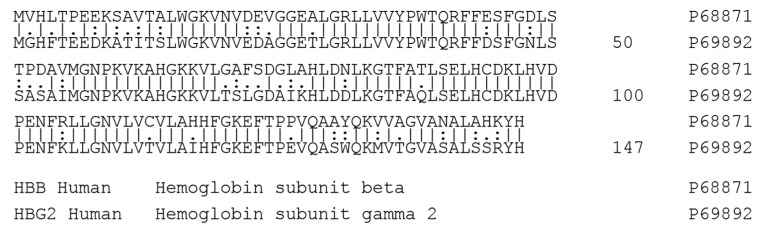
Alignment of the β and γ chains, respectively, using the CLUSTAL program [53]. The identity between the two chains is 73.5%.

During the imprinting procedure, HbF was chosen as the template molecule. While the overall quaternary structure of HbF closely resembles that of HbA, notable differences in 39 residues between the β and γ chains contribute to significant distinctions in the properties between HbA and HbF (see Figure 2). Compared to HbA, HbF demonstrates heightened stability and oxygen affinity, as well as augmented pseudoperoxidase and nitrite reductase activities [39,43,47,48,49]. Recently, there has been a burgeoning interest in the investigation of HbF, particularly regarding its potential utility as a foundational material for the further development of HBOCs [39,50,51,52].

**Figure 3:**
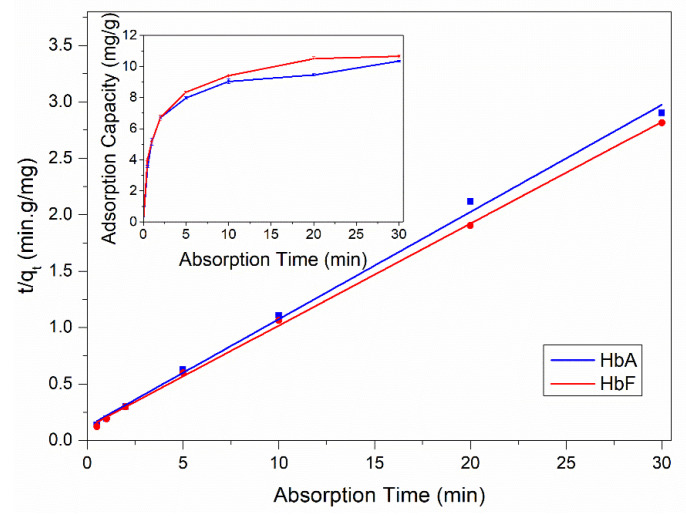
Analysis of the obtained kinetic binding data using a pseudo-second-order kinetic model. The inset shows the kinetics of HbA and HbF binding with 5 mg of MIP particles. The initial Hb concentration was 6 μM.

The MIPs developed in this study feature surface-exposed molecular binding sites, indicating that the MIP beads were anticipated to exhibit rapid protein-binding kinetics due to their open diffusion channels. To verify this assumption, we conducted kinetic binding experiments using both recombinant HbA and HbF. As illustrated in the inset of Figure 3, the HbF-MIPs achieved 90% adsorption equilibrium within 10 min for both HbA and HbF. This rapid adsorption kinetics underscored the excellent mass transport capabilities of the produced imprinted particles, thus addressing some limitations of conventional imprinted particles that typically necessitate prolonged incubation periods (several hours) to attain the binding equilibrium [54,55]. To delve deeper into the adsorption kinetics, we applied a pseudo-second-order kinetic model to fit the experimental data, yielding the initial sorption rate (V_0_) and the equilibrium uptake (Q_e_) (refer to Figure 3 and Table 1). As indicated in Table 1, the adsorption data exhibited a good fit with this model, boasting high correlation coefficients exceeding 0.99 for both HbA and HbF. Under the specified incubation conditions, the HbF-MIP demonstrated marginally higher Q_e_ and V_0_ values for HbF.

**Figure 4:**
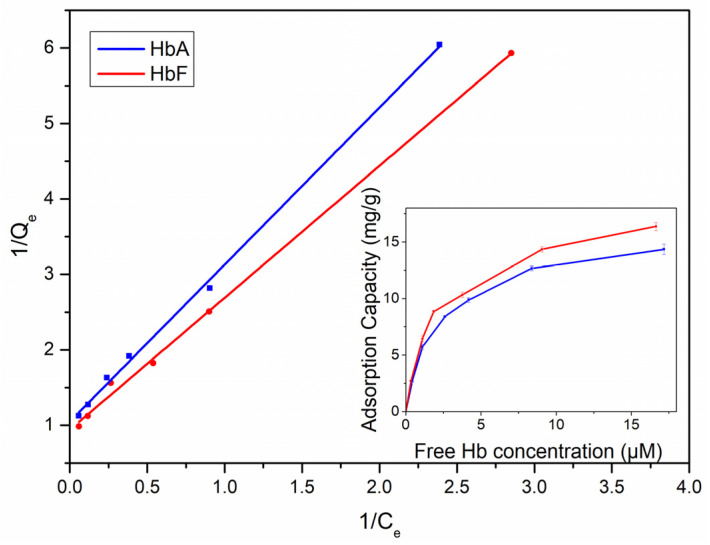
Analysis of the equilibrium adsorption data using the Langmuir model. The inset shows the equilibrium HbA and HbF binding with 5 mg of MIP particles. The incubation time was fixed at 10 min for equilibrium adsorption.

We also investigated the equilibrium adsorptions of the particles for both HbA and HbF, employing the Langmuir equation to analyze the data and extract quantitative insights (refer to Figure 4 and Table 2). As depicted in Table 2, the adsorption isotherms exhibited a good fit with the Langmuir model. The saturation capacities of HbA and HbF were determined to be 15.4 and 17.1 mg/g polymer, respectively. Additionally, the association constant (K_a_) for HbF (×10^5^ M^−1^) was observed to be slightly higher than that for HbA.

**Figure 5:**
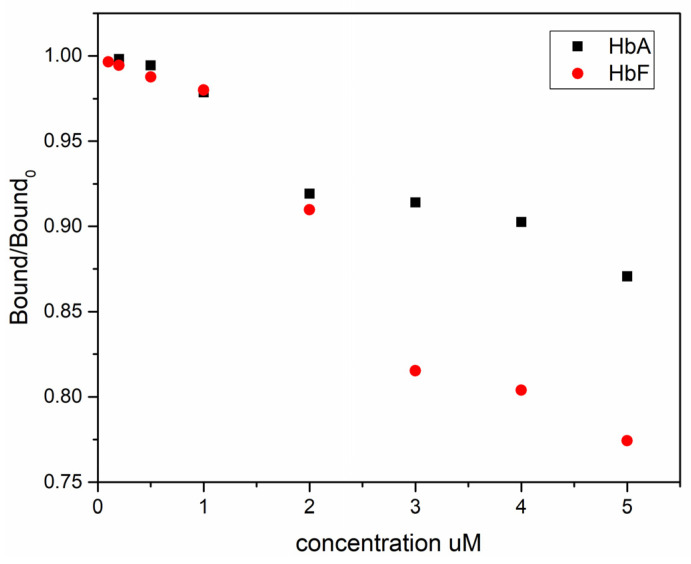
Displacement of GFP-HbF from 5 mg of MIP particles by HbA and HbF. Bound and Bound_0_ are the amount of the bound GFP-HbF measured in the presence and absence of competing HbA/HbF, respectively.

To assess the influence of the template structure on target binding, we conducted competitive binding experiments, where wildtype HbA and HbF were employed as competing molecules to displace a GFP-HbF reporter protein from the imprinted polymer particles. GFP exhibits intense green fluorescence under light in the blue to ultraviolet range, with an excitation peak at 390 nm and a primary emission peak at 508 nm [56]. To facilitate the evaluation of Hb molecules’ performance and interactions in both cell-free environments and biological test systems, a GFP molecule was genetically fused to the N-terminus of HbF and expressed in *E. coli*. Due to the hydrophobic surface of GFP and its increased size, the binding capacity of GFP-HbF onto the MIP particles was considerably lower than that of both HbA and HbF. In theory, HbF should serve as the most potent inhibitor for the binding of GFP-HbF to the specific sites. Indeed, as depicted in Figure 5, compared to HbA, HbF significantly outperforms when competing with GFP-HbF for binding to the MIP particles.

### 3.3. Selective Capture of Intact Hb in Complex Samples

**Figure 6:**
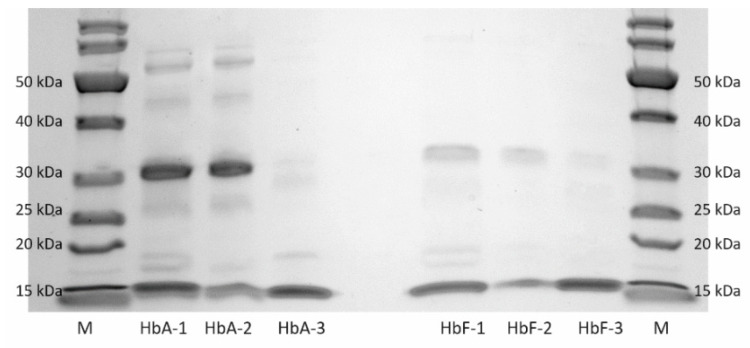
SDS-PAGE of the protein solutions collected from the binding test. Lane M: molecular weight markers; Lane HbA-1/HbF-1: solution of thawed recombinant HbA/HbF before adding the MIPs; Lane HbA-2/HbF-2: solution of unbound recombinant HbA/HbF after depletion with the MIPs; Lane HbA-3/HbF-3: solution of recombinant HbA/HbF eluted from the MIPs.

The remarkable selectivity of the HbF-MIPs proves invaluable for a range of protein separation applications. To showcase their potential, we employed the MIPs to eliminate interfering denatured and aggregated Hb variants from thawed recombinant Hb samples previously stored in a frozen state. Under such conditions, Hb undergoes significant reactivity, leading to several side reactions, including globin chain dimerization, poypeptide misfolding, protein aggregation and heme loss (refer to Figure 6, lanes HbA-1 and HbF-1). Notably, bands at 32 kDa indicate the presence of fused αβ/αγ dimers that can form during storage. Consequently, functional Hb preparations often require purification before use, employing various chromatographic techniques exploiting differences in protein size, physicochemical properties, binding affinity and biological activity [57].

As depicted in Figure 6, the MIP particles demonstrated the ability to selectively capture both normal intact HbA and HbF molecules (with a purity of 98%, lanes HbA-3 and HbF-3) while effectively removing interfering degraded Hb proteins, particularly the fused αβ/αγ dimers, from the solution (lanes HbA-2 and HbF-2). This capability to separate and purify intact Hb from proteins with high structural similarity clearly highlights the high selectivity of the imprinted polymer absorbent, offering practical utility in protein separation and analysis.

### 3.4. Chromatographic Separation of HbA and HbF from E. coli Crude Cell Extracts

The utilization of recombinant means for Hb production offers numerous advantages. Through various protein engineering strategies, the physiological suitability of the Hb protein can be tailored and enhanced to address specific efficacy and toxicity concerns [42,58]. Moreover, mutagenesis techniques can be employed to boost expression yields, thereby facilitating Hb production on a larger scale [52]. Bacterial expression systems are commonly utilized for Hb production, with approximately 60% of all recombinant proteins described in the literature and nearly 30% of currently approved recombinant therapeutic proteins being produced in *E. coli* systems. This preference is attributed to factors such as the low cost of media, rapid growth, ease of handling and extensive understanding of host cell genetics [59].

**Figure 7:**
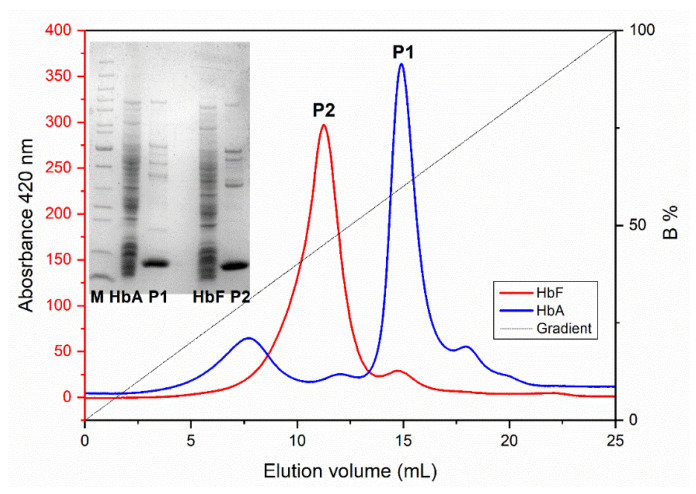
Elution profiles of recombinant HbA and HbF in *E. coli* crude cell extracts from the MIP column using a linear gradient of buffer A (20 mM sodium phosphate buffer, pH 6.0) mixed with buffer B (20 mM sodium phosphate buffer, pH 8.0). The inset is the SDS-PAGE of the peak fractions. Lane M: molecular weight marker; Lane HbA/HbF: the HbA/HbF crude extracts; Lane P1/P2: the elution peaks of HbA/HbF from the MIP column.

In this study, we packed the obtained MIP particles into a HR column and explored the potential of using MIPs as chromatographic resin for the specific isolation of Hb directly from crude cell extracts of *E. coli*. The bacterial production of Hb is complex, often resulting in various misfolded and aggregated polypeptides, large quantities of *E. coli* host cell proteins (HCPs), modified heme and free porphyrin [57]. Initially, solid contaminants were removed through centrifugation or filtration, yielding a “crude extract”. Subsequently, these extracts were directly applied to the MIP column. Figure 7 illustrates the elution profiles of recombinant HbA and HbF from the MIP column, along with the SDS-PAGE analysis of the collected protein solutions. It is evident from the results that the bound HbA/HbF molecules could be eluted from the Hb-MIP column by simply adjusting the pH from 6.0 to 8.0. Notably, when employing a pH gradient, HbA eluted later from the column compared to HbF. Considering the isoelectric point of Hb is approximately pH 6.8, Hb molecules carry a positive charge at pH 6.0 and a negative charge at pH 8.0. Since the β chain possesses two more positively charged residues compared to the γ chain, as depicted in Figure 2, at pH 6.0, the net charge of HbA is slightly higher than HbF. Therefore, a greater pH shift is required to elute HbA from the column. During the dynamic binding process, it is plausible that ionic interactions play a crucial role. SDS-PAGE analysis revealed that most proteins in the crude cell extract did not bind to the imprinted polymer particles, indicating that this one-step purification by the HbF-MIP column resulted in highly purified Hb preparations.

## 4. Conclusions

This study introduces an alternative method for producing HbF-imprinted polymer beads, designed for selective protein separation. By immobilizing HbF on silica nanoparticles, we utilized them as molecular templates to create protein-specific binding sites on the surfaces of cross-linked polymer particles. The resulting imprinted polymer beads demonstrate rapid and selective protein recognition for both recombinant HbA and HbF. The potential of utilizing these MIPs as chromatography resin for protein purification motivates further optimization and scaling up of the MIP synthesis, aiming for efficient downstream processing of recombinant Hb for the development of HBOCs. The present large-scale MIP columns allow preparative isolation of Hb from crude bacterial extracts.

## Figures and Tables

**Table 1:** Summary of the fitted parameters of the adsorption kinetics.

	Equation	Q_e_ (mg g^−1^)	V_0_ (mg g^−1^ min^−1^)	R^2^
HbA	y = 0.0950x + 0.1253	10.53	7.98	0.997
HbF	y = 0.0902x + 0.1168	11.08	8.56	0.999

**Table 2:** Langmuir plot fitting of the equilibrium adsorption on the MIP particles.

	Equation	K_a_ (×10^5^ M^−1^)	Q_max_ (mg/g)	R^2^
HbA	y = 2.0820x + 1.0477	5.03	15.4	0.998
HbF	y = 1.7495x + 0.9413	5.38	17.1	0.998

## Data Availability

The original contributions presented in the study are included in the article, further inquiries can be directed to the corresponding author.
